# Protocol for studying the efficiency of ChemoCalc software in helping patients to understand drug treatment costs for breast cancer: A multicenter, open-label, randomized phase 2 study

**DOI:** 10.1016/j.conctc.2021.100739

**Published:** 2021-02-11

**Authors:** Sayaka Kuba, Hiroki Moriuchi, Kosho Yamanouchi, Kenichiro Shibata, Hiroshi Yano, Masahiro Oikawa, Shigeto Maeda, Xiangyue Meng, Michi Morita, Toshiko Hatachi, Ryota Otsubo, Megumi Matsumoto, Junya Miyamoto, Kengo Kanetaka, Hideki Taniguchi, Takeshi Nagayasu, Susumu Eguchi

**Affiliations:** a Department of Surgery, Nagasaki University Graduate School of Biomedical Science, Nagasaki, Japan; b Department of Surgery, National Hospital Organization Saga Hospital, Saga, Japan; c Department of Surgery, National Hospital Organization Nagasaki Medical Center, Nagasaki, Japan; d Department of Surgery, Japanese Red Cross Nagasaki Genbaku Hospital, Nagasaki, Japan; e Department of Surgical Oncology, Nagasaki University Graduate School of Biomedical Science, Nagasaki, Japan; f Department of Breast Surgery, Oikawa Hospital, Fukuoka, Japan; g Clinical Research Center of Nagasaki University Hospital, Nagasaki, Japan

**Keywords:** Breast cancer, Clinical trials communication, Oncology

## Abstract

Survival of patients with breast cancer can be prolonged by treatment with drugs, particularly new molecular-targeted drugs. However, these agents can be expensive and such treatments can be “an economic burden.” In this ongoing trial, we aim to assess the usefulness of ChemoCalc, a software package for calculating drug costs, to help patients understand the financial outlays. In this multicenter, randomized controlled phase 2 trial, 106 patients with advanced breast cancer will be assigned to either the “ChemoCalc” or “Usual Explanation” group. Treatment using ChemoCalc will be discussed with patients in the ChemoCalc group, whereas standard treatments, without using ChemoCalc, will be discussed with patients in the Usual Explanation group. Subsequently, the participants will decide the treatment and complete a five-grade evaluation questionnaire; those in the Usual Explanation group will receive information about ChemoCalc. Investigators will report if patients subsequently decide to change treatments. The primary endpoint will be the scores of two key questions compared between the groups: “Did you understand the cost of treatment in today's discussion?” and “Do you think the cost of treatment is important in choosing a treatment?“. The secondary endpoints will be to compare discrepancies between treatments recommended by physicians and those selected by patients, the time required for discussion, other questionnaire factors, and the relationship between Comprehensive Score for Financial Toxicity tool and treatment selection. This will be the first randomized controlled trial to assess the efficacy of software to help patients understand drug cost estimates and whether it subsequently affects treatment choice. This study will be conducted according to the CONSORT statement. All participants will sign a written consent form. The study protocol was reviewed and approved by the Clinical Research Review Board of Nagasaki University (19070801). The protocol (version 1) was designed and will be conducted in accordance with the Declaration of Helsinki (1964) and the Ethical Guidelines for Medical and Health Research Involving Human Subjects (2017). The findings will be disseminated through scientific and professional conferences, and in peer-reviewed journals.

**Trial registration:**

UMIN Clinical Trials Registry, UMIN000039904. https://upload.umin.ac.jp/cgi-open-bin/ctr/ctr_view.cgi?recptno=R000041968

## Highlights

• We aim to verify the efficiency of the drug cost estimation software ChemoCalc
• We will demonstrate whether knowing drug costs will change treatment choice
• We will elucidate whether ChemoCalc will enable patient–physician discussions

## 1 Introduction

More than 90,000 breast cancer cases are reported in Japan annually, with nearly 15,000 associated deaths [1]. Advanced breast cancer is treatable but not curable, and the goals of patient care are to improve symptoms, support quality of life (QOL), and prolong survival time [2]. Subtype-specific treatments, especially emerging new drugs, have gradually increased patient survival after relapse [3]. As a new targeted therapy for patients with hormone receptor-positive breast cancer, cyclin-dependent kinase (CDK) 4/6 inhibitor administered in combination with fulvestrant significantly delays deterioration in patient-reported outcomes of QOL and pain compared with fulvestrant alone [4]. However, such novel drugs can be highly expensive. “Financial toxicity” is an emerging problem, in which high out-of-pocket medical costs are associated with a decreased QOL, delayed or forgone care, or a combination of these, increasing the risk of adverse health outcomes [5,6]. Although approximately 50% of doctors and patients want to discuss out-of-pocket costs related to treatment, only 19% of the patients talk to their doctors about costs [7]. Approximately 60% of patients who have these discussions eventually decide to reduce their out-of-pocket costs. A pilot survey of oncologists reported that 42% of them assessed patient financial distress, but only 20% felt that they could intervene in financial toxicity [8].

One major barrier to patient–physician cost communication is a lack of accurate and accessible information on costs, especially out-of-pocket costs [9]. To improve price transparency, various web-based resources have been developed [10]. Eviti ADVISOR compares expected costs, outcomes, and toxicities of different treatments [11]. DrugAbacus provides current and abacus prices in an attempt to estimate the “value-based process” [12]. However, the variability in health insurance and the segmented care system make it extremely difficult to estimate out-of-pocket costs.

Japan has an excellent public health insurance system and all citizens are eligible for enrollment. The out-of-pocket costs of patients are determined based on their age (≥70 or <70 years) and income, with copayments at 10%, 20%, or 30% of the full medical costs [13]. The maximum out-of-pocket costs are 8,000–252,600 yen per month depending on the income tiers. If patient costs exceed the limit for more than 3 months in a year, the copayment is further reduced.

ChemoCalc (Nippon Chemiphar, Tokyo, Japan; https: https://www.nc-medical.com/chemocalc_web/app/) is a free downloadable software for calculating drug costs, including the costs of endocrine therapy, chemotherapy, and supportive therapies such as antiemetics. The software was created to assist doctors and pharmacists in estimating the cost of drug treatments for patients with breast cancer. We reported its usefulness in assisting patients to understand drug costs in a pilot study [14]. In this randomized controlled phase 2 trial, we aim to verify the efficiency of ChemoCalc for women with metastatic breast cancer.

## 2 Methods and analysis

### 2.1 Study design

**Fig. 1:**
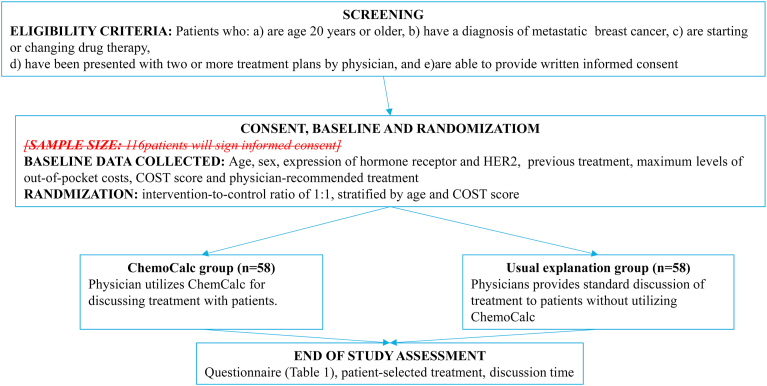
Study flow diagram.

In this ongoing multicenter, randomized controlled open-label phase 3 trial, we aim to evaluate the effect of ChemoCalc on the understanding of patients regarding medical costs. A study flow diagram is shown in Fig. 1. This study was approved by the Nagasaki University Hospital Clinical Research Ethical Committee (registration number 19070801), Japan. Any changes to the trial protocol will be communicated to all investigators, ethics committees, and the trial registry.

### 2.2 Eligibility criteria

Eligible individuals are patients aged ≥20 years and diagnosed with metastatic breast cancer, who are starting or changing a drug therapy, have two or more treatment plans presented by the attending physician, and can provide written informed consent. Both men and women can participate in this study. Patients with dementia will be excluded because they would not be able to complete a questionnaire. Patients with no out-of-pocket costs will be excluded.

### 2.3 Recruitment, setting, and informed consent

Patients planning treatment for metastatic breast cancer are being recruited from Nagasaki University, National Hospital Organization Nagasaki Medical Center, National Hospital Organization Saga Hospital, and Japanese Red Cross Nagasaki Genbaku Hospital from July 31, 2019 onward. All participants are dispensed a participant information sheet and requested to provide their written consent. Participants can withdraw from the study at any time without having to provide a reason.

### 2.4 Randomization

Patients will be allocated to groups using a stratified block randomization method. Each investigator will assign participants to the intervention or control group using a centralized, remote computer-generated randomizer built in Research Electronic Data Capture electronic data capture system (REDCap). The stratification factors are age (<50 years and ≥50 years) and grade of Comprehensive Score for Financial Toxicity (COST) tool (grade 0 and grade 1 or higher) [15,16]. There will be an intervention-to-control ratio of 1:1.

### 2.5 Assessment schedule and treatment

**Fig. 2:**
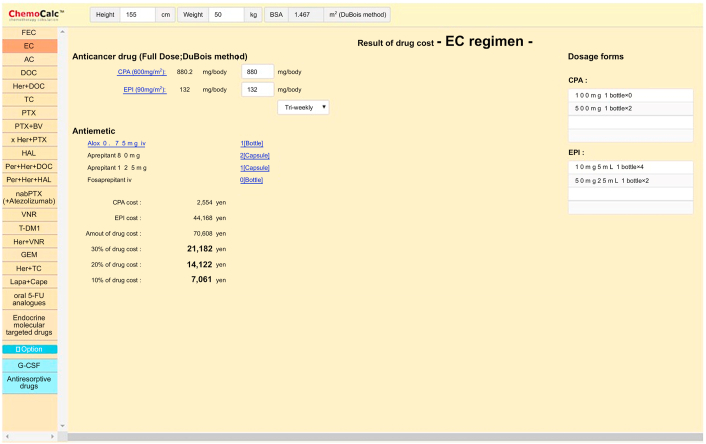
Example of ChemoCalc.

After consenting to participate in the study, patients will discuss treatment, determine their treatment plan, and respond to a five-point questionnaire about their decision-making process (Table 1). In the discussion phase, patients will be randomly assigned to one of the two groups, and those in the ChemoCalc group will discuss treatment with their doctors using ChemoCalc. Body surface area (BSA) will be calculated by inputting the height and weight of patients to ChemoCalc (Fig. 2a) [14]. After selecting a treatment regimen, ChemoCalc will be used to calculate the dose and cost of the drug, at 10%, 20%, and 30% (Fig. 2b) [14]. In the Usual Explanation group, doctors will discuss treatment with patients without using ChemoCalc. After the participants in the Usual Explanation group complete the five-grade evaluation questionnaire, they will receive information about ChemoCalc. Investigators will also report changes in treatment decisions made by patients after the acquisition of this information. The study period is from the time when consent is obtained until treatment drug is decided.

**Table 1:** Questionnaire items.

1. What is your profession?
2. What is your marital status?
3. Do you have any children?
4. Do you have commercial medical insurance?
5. Did you understand drug costs in today's discussion?
6. Do you think drug cost is important when deciding on treatment?
7. Do you think you were able to independently choose your treatment during today's discussion?
8. Are you satisfied with your treatment choice based on today's discussion?
9. Did you discuss the financial burden of treatment with your doctor during today's discussion?
10. Do you think patients should talk more to health care professionals about the financial burden of treatment?
11. With whom do you want to discuss the financial burden of treatment?
12. Please tell us what concerns you currently have about your treatment.
13. (ChemoCalc group only) Was ChemoCalc easy to understand?
14. Circle the numbers that best describe the importance of choosing a treatment. The lower the score, the lower the importance.

Drug efficacy	0	1	2	3	4
Drug-related adverse effects	0	1	2	3	4
Drug cost	0	1	2	3	4
Number of visits to hospital	0	1	2	3	4
Your doctor strongly recommends treatment	0	1	2	3	4

### 2.6 Trial endpoints

• Primary endpoint

The primary endpoint will be the comparison of scores of two key questions in the questionnaire between the two groups: “Did you understand the drug cost in today's discussion?” and “Do you think the drug cost is important when deciding on treatment?“. The questions will be rated on a five-point scale, with 1 being “not at all” and 5 being “very much."

• Second'ary endpoints
  1. A comparison of the scores of other questions in the questionnaire (Table 1).
  2. A comparison of the discrepancy between physician-recommended and patient-selected treatments between the two groups (the patient-selected treatment in the Usual Explanation group is that selected before providing ChemoCalc information). Before discussion with patients, the physicians will register treatment plan ranking using REDCap. Any discrepancy will be judged based on whether the physician's first recommended treatment is selected. If there is a discrepancy, we will investigate whether the treatment choice is more expensive, equal, or less expensive than the first recommended treatment.
  3. A comparison of discussion time between the two groups. Discussion time is defined as the duration of one interview to discuss the treatment plans.
  4. The relationship between COST grade and patient-selected treatment. We will categorize the treatments chosen by patients and the first recommended treatments by the physicians into groups with and without discrepancies. In both groups, we will divide treatments selected by patients into three groups: higher, equivalent, and lower than the first recommended treatment by the physician. We will then examine whether it is related to the COST grade.
  5. The relationship between the upper limit of out-of-pocket costs and patient-selected treatment. We will divide the treatments chosen by patients and the first recommended treatments by the physicians into groups with and without discrepancies. In both groups, we will divide treatments selected by patients into three groups: higher, equivalent, and lower than the first recommended treatment by the physician. We will then examine whether it is related to the upper limit of out-of-pocket costs.

### 2.7 Sample size

Based on a previous study [14], the median (interquartile range) scores of the questionnaire items will be as follows: “Did you understand the drug cost?” will be 2.5 (1.0–4.0) and 5.0 (4.25–5.00) in the Usual Explanation and ChemoCalc groups, respectively. Whereas, the corresponding scores for “Do you think the drug cost is important when deciding on treatment?” will be 3.0 (3.0–4.5) and 5.0 (4.25–5.0), respectively. At 90% power and a 0.025 significance level, analysis using Mann–Whitney *U* test with Bonferroni correction indicated that the trial would require 47 patients in each group. Therefore, considering a 10% potential withdrawal rate, we aim to enroll 106 patients (53 per group).

### 2.8 Data management

Registration of patients and data management will be carried out using REDCap. Only the principal investigator, subinvestigators, and research collaborators, registered at the research secretariat will be able to access REDCap to enter and modify data. The original questionnaire will be submitted to Nagasaki University Hospital and the data will be provided a study code; blinded data will be analyzed. All data will be discarded 5 years after completion of the study.

### 2.9 Statistical analyses

Analyses of the primary and secondary outcomes will be based on intent-to-treat and full analysis sets, which will include withdrawal from the study. Patients' clinicopathological features will be compared between the intervention and control groups. Variables will be presented as frequency (for categorical variables) and as median and interquartile range (for quantitative variables). Fisher's exact test will be used to assess associations between categorical variables, and *[Wilcoxon's rank sum test]* Mann–Whitney *U* test will be used to assess quantitative variables. All statistical analyses will be performed using SAS, EZR, and JMP software programs.

### 2.10 Study status

Patient enrollment started in July 2019 and 36 patients had been recruited as of April 2020, and the recruitment is ongoing.

## 3 Discussion

This will be the first randomized control trial to verify the efficacy of the drug cost estimation software ChemoCalc in assisting patients with advanced breast cancer to select treatments that are more cost effective. Our findings will help identify treatment choice changes under improved cost communication. The American Society of Clinical Oncology established a Cost of Care Task Force in 2009, which developed a Guidance Statement on the Cost of Cancer Care [17]. It stated that patient–physician discussions regarding cost are an important component of high-quality care and that educational and support tools for oncology providers are needed to discuss costs with patients and help guide decision making. Although all institutions participating in this study are certified by the Japanese Breast Cancer Society and all investigators specialize in breast cancer, doctors are specifically educated regarding cost communication. We hope to demonstrate that ChemoCalc is a useful tool for informing patients and explaining drug treatment options.

Patients’ impression regarding the importance of drug cost in making treatment decisions based on high out-of-pocket costs may be associated with medication nonadherence [18]. Among patients with chronic myelogenous leukemia in Japan, approximately 30% considered treatment discontinuation because of treatment-related financial burden [19]. We believe that by improving cost communication between patients and their doctors, patients will be able to judge whether a treatment is economically feasible in advance, ensuring adherence and avoiding treatment discontinuation. Therefore, we aim to assess whether patients consider drug costs to be important as a key endpoint.

Some limitations of the study should be noted. First, because Japan's medical insurance system is unique, the results of this study can only be applied for cost communication between Japanese doctors and patients. However, an understanding of how pre-treatment cost communication affects patient treatment choices should benefit all physicians and patients. Second, our study does not list specific guidelines for these discussions, other than the inclusion or exclusion of ChemoCalc. Few doctors may discuss drug cost during routine medical appointments, but others may not. Because our aim is to investigate whether ChemoCalc changes patients' understanding of drug costs regardless of their assigned physician, we did not standardize the methods by which physicians will present or explain drug cost issues.

In conclusion, in the present study, we will assess a strategy of drug cost communication in Japan and characterize the importance of cost communication in patients with advanced breast cancer.

## Funding

This research did not receive any specific grant from funding agencies in the public, commercial, or not-for-profit sectors.

## Authors’ contributions

SK, KY, HM, KS, MM2, MO, and SM are responsible for conceiving and designing the trial, planning analysis, and collecting data, and will be managing the recruitment and treatment of patients. XM, TH, RO, and HY will participate in collecting data and managing patient recruitment and treatment. JM is responsible for sample size determination and allocation. KK, HT, TN, and SE are responsible for planning data analysis and analyzing the data obtained from the trial. MM1 will access the final trial dataset and analyze the data. All authors contributed and approved the final version of the manuscript for publication.

## Declaration of competing interest

The authors declare that they have no competing interests.
