# Supplementary material for: Protocol for studying the efficiency of ChemoCalc software in helping patients to understand drug treatment costs for breast cancer: A multicenter, open-label, randomized phase 2 study
Source: Contemp Clin Trials Commun. 2021 Feb 11;21:100739. doi: 10.1016/j.conctc.2021.100739 (PMC7921475; doi:10.1016/j.conctc.2021.100739)
Supplement: Multimedia component 1 [file mmc1.docx]

install.packages("exactRankTests", repos="http://cran.ism.ac.jp/")

library(exactRankTests)

makeData <- function (sampleSize){

probACat1 <- c(0.4, 0.1, 0.1, 0.3, 0.1)

probBCat1 <- c(0, 0, 0.1, 0.3, 0.6)

probACat2 <- c(0.1, 0.0, 0.6, 0.0, 0.3)

probBCat2 <- c(0.1, 0, 0.1, 0.2, 0.6)

for (i in 1:length(probACat1)) {

if (i == 1) {

accumProbACat1 <- c(probACat1[i])

} else {

accumProbACat1 <- c(accumProbACat1, accumProbACat1[i - 1] + probACat1[i])

}

}

for (i in 1:length(probBCat1)) {

if (i == 1) {

accumProbBCat1 <- c(probBCat1[i])

} else {

accumProbBCat1 <- c(accumProbBCat1, accumProbBCat1[i - 1] + probBCat1[i])

}

}

for (i in 1:length(probACat2)) {

if (i == 1) {

accumProbACat2 <- c(probACat2[i])

} else {

accumProbACat2 <- c(accumProbACat2, accumProbACat2[i - 1] + probACat2[i])

}

}

for (i in 1:length(probBCat2)) {

if (i == 1) {

accumProbBCat2 <- c(probBCat2[i])

} else {

accumProbBCat2 <- c(accumProbBCat2, accumProbBCat2[i - 1] + probBCat2[i])

}

}

randACat1 <- runif(sampleSize,0,1)

randBCat1 <- runif(sampleSize,0,1)

randACat2 <- runif(sampleSize,0,1)

randBCat2 <- runif(sampleSize,0,1)

orderListACat1 <- list()

orderListBCat1 <- list()

orderListACat2 <- list()

orderListBCat2 <- list()

for (i in 1:length(accumProbACat1)) {

if (i == 1) {

if (length(which(randACat1 <= accumProbACat1[i])) > 0) {

orderListACat1[i] <- list(which(randACat1 <= accumProbACat1[i]))

#browser()

} else {

orderListACat1[i] <- list(NULL)

}

} else {

if (length(which(accumProbACat1[i - 1] < randACat1 & randACat1 <= accumProbACat1[i])) > 0) {

orderListACat1[i] <- list(which(accumProbACat1[i - 1] < randACat1 & randACat1 <= accumProbACat1[i]))

} else {

orderListACat1[i] <- list(NULL)

}

}

}

for (i in 1:length(accumProbBCat1)) {

if (i == 1) {

if (length(which(randBCat1 <= accumProbBCat1[i])) > 0) {

orderListBCat1[i] <- list(which(randBCat1 <= accumProbBCat1[i]))

#browser()

} else {

orderListBCat1[i] <- list(NULL)

}

} else {

if (length(which(accumProbBCat1[i - 1] < randBCat1 & randBCat1 <= accumProbBCat1[i])) > 0) {

orderListBCat1[i] <- list(which(accumProbBCat1[i - 1] < randBCat1 & randBCat1 <= accumProbBCat1[i]))

} else {

orderListBCat1[i] <- list(NULL)

}

}

}

for (i in 1:length(accumProbACat2)) {

if (i == 1) {

if (length(which(randACat2 <= accumProbACat2[i])) > 0) {

orderListACat2[i] <- list(which(randACat2 <= accumProbACat2[i]))

#browser()

} else {

orderListACat2[i] <- list(NULL)

}

} else {

if (length(which(accumProbACat2[i - 1] < randACat2 & randACat2 <= accumProbACat2[i])) > 0) {

orderListACat2[i] <- list(which(accumProbACat2[i - 1] < randACat2 & randACat2 <= accumProbACat2[i]))

} else {

orderListACat2[i] <- list(NULL)

}

}

}

for (i in 1:length(accumProbBCat2)) {

if (i == 1) {

if (length(which(randBCat2 <= accumProbBCat2[i])) > 0) {

orderListBCat2[i] <- list(which(randBCat2 <= accumProbBCat2[i]))

#browser()

} else {

orderListBCat2[i] <- list(NULL)

}

} else {

if (length(which(accumProbBCat2[i - 1] < randBCat2 & randBCat2 <= accumProbBCat2[i])) > 0) {

orderListBCat2[i] <- list(which(accumProbBCat2[i - 1] < randBCat2 & randBCat2 <= accumProbBCat2[i]))

} else {

orderListBCat2[i] <- list(NULL)

}

}

}

#browser()

for (i in 1:length(accumProbACat1)) {

randACat1[unlist(orderListACat1[i])] <- i

#browser()

}

for (i in 1:length(accumProbBCat1)) {

randBCat1[unlist(orderListBCat1[i])] <- i

#browser()

}

for (i in 1:length(accumProbACat2)) {

randACat2[unlist(orderListACat2[i])] <- i

#browser()

}

for (i in 1:length(accumProbBCat2)) {

randBCat2[unlist(orderListBCat2[i])] <- i

#browser()

}

wilcoxData <- cbind(randACat1, randBCat1, randACat2, randBCat2)

return(wilcoxData)

}

calcKubaPower <- function(trial, sampleSize, seed) {

set.seed(seed)

res <- matrix(ncol=2)

for (i in 1:length(sampleSize)) {

pvalCat1 <- c()

pvalCat2 <- c()

for (j in 1:trial) {

wilcoxData <- makeData(sampleSize[i])

wilcoxData <- data.frame(wilcoxData)

resCat1 <- wilcox.exact(as.integer(wilcoxData$randACat1),as.integer(wilcoxData$randBCat1),paired=F)$p.value

resCat2 <- wilcox.exact(as.integer(wilcoxData$randACat2),as.integer(wilcoxData$randBCat2),paired=F)$p.value

pvalCat1 <- c(pvalCat1, resCat1)

pvalCat2 <- c(pvalCat2, resCat2)

}

significantRes <- c()

for (k in 1:trial) {

if (pvalCat1[k] < 0.025 & pvalCat2[k] < 0.025) {

significantRes <- c(significantRes, 0)

} else {

significantRes <- c(significantRes, 1)

}

}

res <- rbind(res, c(sampleSize[i], 1-(sum(significantRes)/trial)))

print(res)

}

#browser

res

}
